# Acute Pancreatitis After Initiating Dulaglutide in a Patient Previously Treated With a DPP-4 Inhibitor: Case Report From Palestine

**DOI:** 10.1155/crgm/9918202

**Published:** 2025-11-28

**Authors:** Alisse Nasser, Hosniyeh Ladadweh, Raed Madia, Ahmad Dalashi, Adnan Wahdan, Abdullah Alawi

**Affiliations:** ^1^Pharmacy Department, Faculty of Pharmacy, Nursing and Health Professions, Birzeit University, Birzeit, State of Palestine; ^2^Internal Medicine Specialist, Palestine Medical Complex, Ministry of Health, Ramallah, State of Palestine

**Keywords:** acute pancreatitis, glucagon-like peptide-1 (GLP-1), obesity conclusion, type 2 diabetes (T2DM)

## Abstract

**Background:**

Dulaglutide, a glucagon-like peptide-1 (GLP-1) agonist, is utilized for the management of type 2 diabetes and obesity, typically administered subcutaneously at a dosage of 0.75 mg/0.5 mL once weekly, with adjustments made as needed. While typically well tolerated, it may induce infrequent yet severe adverse effects, including acute pancreatitis.

**Case Presentation:**

In this case report, we present the first documented case of GLP-1-induced pancreatitis in Palestine. A 62-year-old female Palestinian patient with a history of type 2 diabetes complicated with nephropathy, hypertension, dyslipidemia, and cardiac catheterization three years prior without stenting. The patient developed acute pancreatitis following the initiation of 1.5 mg of a GLP-1 receptor agonist after 4 months of therapy. The patient experienced upper abdominal pain radiating to the back, nausea, and alternating diarrhea and constipation for 5 days. Laboratory investigations revealed elevated serum amylase and lipase levels, and imaging studies confirmed signs of acute pancreatitis. After the GLP-1 agonist was discontinued, the patient's symptoms improved, and she fully recovered.

**Conclusion:**

GLP-1 receptor agonists are advantageous for the management of diabetes and obesity but may precipitate acute pancreatitis in predisposed individuals. Clinicians should be aware of this potential risk and promptly investigate any patient who presents with signs of acute abdominal pain during GLP-1 therapy.

## 1. Introduction

Acute pancreatitis is defined by the American Gastroenterological Association as sudden inflammation of the pancreas that can be life-threatening [1]. The pathophysiology of acute pancreatitis is based on the premature activation of the enzymes zymogen and trypsinogen, which cause local pancreatic destruction and the activation of the inflammatory cascade, resulting in systemic inflammatory response syndrome (SIRS), which is frequently associated with acute pancreatitis [2]. The inflammatory process can be induced by gallstones, which account for 40%–70% of acute pancreatitis cases [2], alcohol consumption, where chronic and binge drinking can cause pancreatic injury and inflammation [3], and certain medications, such as azathioprine and corticosteroids, which have been linked to pancreatitis [4].

Diagnosis involves clinical evaluation, imaging studies, and laboratory tests. Elevated blood amylase and lipase levels are common signs, and imaging modalities such as CT can detect necrotizing pancreatitis [5]. The Ranson criteria and APACHE II score are commonly used to determine the severity of acute pancreatitis [6]. The management of acute pancreatitis focuses on supportive care and addressing the underlying cause. Key components include fluid resuscitation, prompt intravenous (IV) fluid administration to prevent complications [7], nutritional support, in which early enteral feeding is preferred over parenteral nutrition, as it may reduce the risk of infection and other complications [8], and pain management; opioids are often used to control severe pain, although they must be administered judiciously [9].

Acute pancreatitis can lead to various complications, including pancreatic necrosis. This serious condition can lead to sepsis and multiorgan failure [10], pseudocysts, fluid collections that may require surgical intervention if symptomatic [11], and chronic pancreatitis, which is a result of recurrent episodes leading to chronic inflammation and loss of function [12].

Glucagon-like peptide-1 receptor (GLP-1) agonists are a class of medications that are mainly used to treat type 2 diabetes mellitus (T2DM) and, in some cases, for obesity management, but their role in type 1 DM is currently not well established [13]. After the ingestion of a certain meal containing carbohydrates, proteins, or fat, gastrointestinal peptides such as GLP-1 are released from the small intestine and bind to its receptor, which is found in various tissues, including the pancreas (mainly beta cells and the pancreatic ducts), leading to the secretion of insulin [14]. Typically, the half-life of natural GLP-1 is approximately 1–2 min since it is degraded by the enzyme dipeptidyl peptidase-4 (DPP-4). In T2DM patients, GLP-1 secretion is impaired following a meal, leading to impaired insulin secretion [14].

Compared with natural GLP-1, synthetic GLP-1 has advantages since it is resistant to the effect of the DPP-4 enzyme, allowing increased glucose-dependent insulin release. Exenatide, liraglutide, dulaglutide, and semaglutide are examples of GLP-1 agonists. According to the ADA guidelines, GLP-1 agonists are recommended for individuals with type DM, especially if the patient has or is at high risk of atherosclerotic cardiovascular disease (ASCVD) or has chronic kidney disease (CKD), especially if no albuminuria is present. These agents can be used in combination with metformin or other oral glucose-lowering therapies. They are mainly used when lifestyle interventions and metformin fail to control glucose or when contraindications to metformin exist. Additionally, they have the advantage of causing no hypoglycemia on their own [15].

Dulaglutide is a subcutaneous (SUBQ) GLP-1 agonist approved by the FDA in 2014 [16]. Initially, 0.75 mg of SUBQ should be given once weekly for 4–8 weeks, and the dose may be increased to 1.5 mg for another 4 weeks if an additional glucose-lowering effect is needed. If further glycemic control is needed, the dose may be increased to 3 mg once weekly up to a maximum of 4.5 mg after taking the 3-mg dose for 4 weeks. In cases of renal or hepatic impairment, no dose adjustment is needed. The side effects of dulaglutide include acute kidney injury, diabetic retinopathy, and gastrointestinal symptoms such as diarrhea, nausea, and vomiting, medullary thyroid carcinoma, and pancreatitis [14].

Acute pancreatitis has been reported with the use of dulaglutide, especially if the patient has additional risk factors, such as a prior history of pancreatitis, hypertriglyceridemia, cholelithiasis, alcohol use, and obesity. The hypothesized mechanism of pancreatitis is possibly hyperplasia of pancreatic cells due to GLP-1 stimulation, leading to increased pancreatic weight and pressure, which eventually cause acute or chronic inflammation [14].

## 2. Case Presentation

A 62-year-old female patient with a 5-day history of epigastric pain presented to the emergency department. The pain was constricting in nature, continuous, and radiated to the back. It was accompanied by nausea, reduced oral intake, and a history of weight loss. The patient had a medical history of T2DM for 22 years, hypertension for 15 years, and dyslipidemia for 10 years. Her chronic medications included enalapril (20 mg once daily), bisoprolol (5 mg once daily), atorvastatin (40 mg once daily), glimepiride (3 mg once daily), metformin (1,000 mg once daily), sitagliptin (50 mg once daily), and dulaglutide (1.5 mg once weekly). The GLP-1 receptor agonist (dulaglutide) and DPP-4 inhibitor (sitagliptin) therapies were initiated 4 months prior to presentation. The patient denied alcohol use, smoking, or the consumption of other drugs.

Upon examination, the patient's blood pressure was 112/67 mmHg, and her temperature was 36.8°C, which was normal. The physical findings included dehydrated skin and abdominal obesity. The abdomen was soft and lax, with moderate epigastric tenderness and diminished bowel sounds.

**Figure 1:**
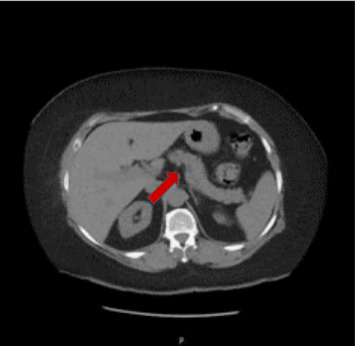
CT scan showing pancreatic edema and peripancreatic fat stranding.

The laboratory results revealed a lipase level of 1899 U/L (normal < 160 U/L), an amylase level of 1,219 U/L (normal < 100 U/L), a creatinine level of 1.82 mg/dL (normal 0.5–0.9 mg/dL), a blood urea nitrogen (BUN) level of 26 mg/dL (normal 8–23 mg/dL), a C-reactive protein (CRP) level of 107.2 mg/dL, a cancer antigen (CA 19–9) level of 79.4 U/mL (normal < 37 U/mL), a hemoglobin level of 11.6 g/dL (slightly low), a white blood cell (WBC) count of 13.9 K/μL (normal 4.5–11.0 K/μL), a total calcium level of 10.08 mg/dL (normal 8.5–10.2 mg/dL), a random blood sugar (RBS) level of 273 mg/dL, and a hemoglobin A1c level of 9.25% (normal < 6%). Abdominal ultrasonography revealed mild pancreatic enlargement, increased Wirsung duct diameters, and surrounding echogenic fat. The gallbladder was absent (postcholecystectomy), and the visualized portion of the common bile duct was not dilated. Mild hydronephrosis was observed in the left kidney. Computed tomography (CT) confirmed pancreatic edema and peripancreatic fat stranding without necrosis, cyst formation, anatomical abnormalities, or stones, as shown in Figure 1.

The patient was diagnosed with acute pancreatitis and prerenal acute kidney injury. Patients with common etiologies of acute pancreatitis, including metabolic causes (normal serum lipid and calcium levels), trauma, alcohol or toxin exposure, autoimmune pathology, anatomical abnormalities (e.g., pancreas divisum), and infections, were excluded. Furthermore, other potential contributors were also excluded. Regarding her home medications, both statins and enalapril may cause direct pancreatic toxicity and secondary metabolic effects; however, the causal relationship remains controversial. Furthermore, the patient was maintained on these medications for several years, on long-term stable doses without any prior pancreatic effects or even biochemical disturbances, thus making it unlikely to be the cause of this current admission. Autoimmune pancreatitis was excluded due to the absence of suggestive clinical features, serological markers, or other imaging findings. In conclusion, this finding was attributed to the recent initiation of high-dose GLP-1 receptor agonist and DPP-4 inhibitor therapy.

Dulaglutide was discontinued. The patient was managed with fasting for the first 48 h, parenteral and oral rehydration, and proton pump inhibitor therapy. An insulin sliding scale was initiated for glycemic control. The serum lipase level dropped to 300 U/L and the serum amylase level dropped to 214.3 U/L within 24 h. By day 3, both levels had returned to normal. Blood glucose levels were stabilized with metformin and fast-acting insulin as needed. Renal function improved, with a reduction in the serum creatinine level, and the hemoglobin level gradually increased to 12.2 g/dL.

The patient experienced significant clinical improvement by the second day, with resolution of pain and nausea. At the 1-month follow-up, she had normal blood glucose levels, normalized biochemical markers, and remained asymptomatic.

## 3. Discussion and Conclusion

Dulaglutide is a long-acting synthetic GLP-1 agonist. It binds to GLP-1 receptors, mainly in the pancreas, to induce glucose-dependent insulin secretion and delays gastric emptying, leading to improved satiety [14].

Our patient was on 1.5 mg of dulaglutide subcutaneously (SC) once weekly for 4 months. Initially, the patient was started on 1.5-mg SC, which is a higher dose. The first dose should be 0.75-mg SC, then titrated based on the response. However, it was not known why the patient was started on 1.5 mg initially rather than 0.75 mg and titrated up. Possible causes may stem from insufficient knowledge about the dosing of dulaglutide and its titration process, which could be classified as a drug-related problem affecting the safety of this medication; the initial dose might have contributed to the development of acute pancreatitis due to skipping the titration step. Additionally, the patient had been on oral sitagliptin for 15 years, and sitagliptin is a DPP-4 inhibitor. It inhibits the degradation of GLP-1, leading to its increased effect. Both GLP-1 agonists and DPP-4 inhibitors are considered incretin treatments.

With respect to the safety profile of GLP-1 agonists reported in a meta-analysis conducted by Yao et al., gastrointestinal side effects such as nausea, vomiting, and diarrhea were reported. Nausea, vomiting, and diarrhea were more common in the treatment group than in the placebo group, with odds ratios (ORs) of 3.13 (95% CI, 2.21–4.44), 2.65 (95% CI, 1.58–4.45), and 2.08 (95% CI, 1.62–2.66), respectively [17].

Compared with GLP-1 agonists, liraglutide has the highest severe rate of gastrointestinal adverse effects (23.31%, *n* = 21,281 reports), whereas dulaglutide has the lowest, with a rate of 12.29% [18].

Research has yielded conflicting results regarding the risk of pancreatitis; the levels of pancreatic enzymes, including amylase and lipase, are known to be elevated in patients on GLP-1 agonists but are usually within the normal ranges. A meta-analysis conducted by Li et al. investigated the risk of pancreatitis due to incretin treatment with either GLP-1 agonists or DPP-4 inhibitors and reported that the rate of pancreatic events was 0.11% (*n* = 33,350) when the patient was receiving one of the treatment options. In addition, no significant difference was observed between the GLP-1 agonist or DPP-4 inhibitor group and the control group, and the risk did not differ according to the type of incretin used. It can be concluded that the risk of pancreatitis due to incretin treatments is considered low, but these results should be used with caution since all the included studies focused on efficacy, and the risk of pancreatitis was extrapolated from these studies; therefore, the generalizability of the findings is limited [19].

Another study conducted by Alenzi et al. [20], which used the postmarketing FDA adverse event reporting system (FAERS), revealed that among 2313 pancreatitis reports, 70.2% were related to GLP-1 agonists, 15% were related only to DPP-4 inhibitors, and 14.7% were related to sodium glucose cotransporters. Additionally, 50.4% of these reports involved female patients, and the highest incidence occurred in those over 50 years old (38.4%) [20].

Causality assessment in pharmacovigilance has emerged as a crucial component for quantitatively predicting the relationship between adverse effects and the drug. Dulaglutide was added to her prior prescription of sitagliptin, and a causality assessment was conducted using the Naranjo scale for adverse drug reactions [21].

For dulaglutide, the score was 6, indicating a probable adverse event. For sitagliptin, the value was −1, indicating a doubtful adverse drug reaction. Furthermore, following the resolution of the patient's pancreatitis, her sitagliptin was resumed, and during her 1-month follow-up, her condition was satisfactory with complete resolution of the pancreatitis, while the dulaglutide was permanently discontinued. Therefore, pancreatitis as an adverse event was attributed to dulaglutide use [21].

The patient was concurrently administered dual incretin therapy (dulaglutide and sitagliptin), potentially heightening the risk of pancreatitis. The concomitant use of a GLP-1 agonist and a DPP-4 inhibitor is not advised in clinical practice, as research indicates that this combination offers no additional benefit. Furthermore, it imposes a pill burden on the patient by increasing the number of medications, resulting in increased costs. This combination is additionally anticipated to increase the risk of adverse events, including pancreatitis [22]. According to the American Diabetes Association (ADA), it is advisable to discontinue the DPP-4 inhibitor when a GLP-1 agonist is considered appropriate, as GLP-1 agonists demonstrate beneficial effects on the cardiovascular system [23].

Currently, the literature contains few case reports of pancreatitis related to dulaglutide use. Further studies are needed to collect more significant data regarding the risk of pancreatitis associated with dulaglutide, which could be accomplished with continued pharmacovigilance.

The treatment for acute pancreatitis is well established in the guidelines, which includes initially clarifying the etiology, which is crucial for planning the treatment approach and preventing recurrence.

Initially, hemodynamic stability should be maintained via fluid resuscitation to achieve a blood pressure of at least 65 mmHg and a urine output of 0.5 mL/kg/h, hence reducing morbidity and mortality [24, 25]. In the latest updates, moderate IV hydration of 1.5 mL/kg/hr is considered sufficient for most patients [26]. Our patient received 4 L of normal saline every 24 h, representing approximately 2.78 mL/kg/hr, which is considered higher than recommended.

Second, patients with acute pancreatitis often have severe and persistent abdominal pain, which is the predominant symptom in almost all patients. Opioids are the preferred options for controlling pain and they are considered effective and generally safe [27]. In terms of nutritional support, oral feeding is reserved for mild cases, whereas enteral support is often preferred for moderate to more severe cases [28, 29]. The patient was maintained on 1 g of paracetamol three times per day, which was generally effective.

Finally, prophylactic antibiotics are not routinely recommended for acute pancreatitis unless an extrapancreatic infection is confirmed [30]. However, numerous systematic reviews have shown that prophylactic antibiotics can reduce mortality and complications from infections [31, 32]. Therefore, if the decision is made to use these antibiotics, broad-spectrum options may be considered. However, despite the recommendation against the use of antibiotics, ciprofloxacin 400-mg IV was prescribed and then changed to 200-mg IV twice daily; its use was not the best option for treatment, and in general, there were no signs of infections requiring antibiotics.

It should be noted certain practices, such as IV hydration and the use of empiric antibiotics, did not adhere to global guidelines. Our patient received aggressive hydration and ciprofloxacin, which exceeded current evidence-based recommendations. Contemporary guidelines advocate for 1.5 mL/kg/hr of fluid resuscitation, which maintains the patient's hemodynamic stability and concurrently minimizes fluid overload. Furthermore, current guidelines discourage the use of prophylactic antibiotics unless infection is confirmed. The deviation from adhering to these standards actually reflects institutional practice rather than a generalized approach. This also clarifies the importance of aligning to the updated guidelines.

In conclusion, this case demonstrates the significance of carefully prescribing GLP-1 agonists, such as dulaglutide, particularly in patients with comorbidities or concurrent antidiabetic agents. Clinicians should start therapy at the lowest effective dose and titrate carefully. To reduce the risk of additional gastrointestinal or pancreatic adverse effects, dual incretin-based therapy should be avoided. It is crucial to keep an eye out for unexpected or persistent abdominal pain because it could indicate major underlying issues, like gallbladder disease or pancreatitis. Maintaining awareness of these potential risks ensures safer, individualized use of GLP-1 receptor agonists in diabetes management.

## Data Availability

The data that support the findings of this study are available from the corresponding author upon reasonable request.
